# The control of allergic rhinitis in real life: a multicenter cross-sectional Italian study

**DOI:** 10.1186/s12948-018-0082-y

**Published:** 2018-02-02

**Authors:** Federica Gani, Carlo Lombardi, Laura Barrocu, Massimo Landi, Erminia Ridolo, Massimo Bugiani, Giovanni Rolla, Gianenrico Senna, Giovanni Passalacqua

**Affiliations:** 1 Allergy Service, Azienda Ospedaliera “San Luigi Orbassano”, Turin, Italy; 2 Departmental Unit of Allergology & Respiratory Diseases, Fondazione Poliambulanza, Brescia, Italy; 3 Primary Care Pediatrician, National Healthcare System, Turin, Italy; 4 Experimental and Clinical Medicine, University of Parma, Parma, Italy; 5 Consultant Physician of Professional Diseases Observatory, Procura della Repubblica, Turin, Italy; 6 Allergology and Immunology, University of Turin, AO Mauriziano, Turin, Italy; 7 Asthma Center and Allergy Unit, Verona University and General Hospital, Verona, Italy; 8 Allergy and Respiratory Diseases, IRCCS San Martino-IST University of Genoa, Genoa, Italy

**Keywords:** Allergic rhinitis, Control, Real-life

## Abstract

**Background:**

Allergic Rhinitis (AR) is a high-prevalence disease. In Europe about 25% of the general population is affected, and in Italy the prevalence is estimated to be 19.8%. The Allergic Rhinitis and its Impact on Asthma (ARIA) international document underlined that the prevalence of severe or refractory or overlapping rhinitis is increasing and represents a non-negligible socio-economic burden. In general, despite the social healthcare costs, allergic rhinitis remains underestimated, not sufficiently controlled and often undertreated.

**Aim of the study:**

In this multi-center Italian observational and prospective study we assessed the control of AR in patients (> 16 years) without previous asthma diagnosis, referred to Allergy Centers.

**Methods:**

Patients of both sexes and older than 16 with rhinitis symptoms and without asthma were studied. A Visual Analogue Scale (VAS) and the CARAT (Control of Allergic Rhinitis and Asthma Test) were used as patient reported outcome. The possible causes of poor control of AR, as per protocol, were assessed accordingly.

**Results:**

We observed 250 patients in a real-life setting: more than 60% of them had an uncontrolled AR, only about 50% used multiple medications, and only a minority were receiving allergen immunotherapy.

**Conclusion:**

This survey, conducted in a real-life setting, confirmed that AR is overall poorly controlled. The VAS assessment well correlates with the structured CARAT questionnaire and with the relevant symptoms of AR.

## Background

Allergic Rhinitis (AR) is a high-prevalence disease. In Europe about 25% of the general population is affected and in Italy, the prevalence is estimated to be 19.8% [1]. The Allergic Rhinitis and its Impact on Asthma (ARIA) international document [2] underlined that severe or refractory or mixed forms of AR are increasing and represent a not negligible socio-economic burden [3]. In addition, it was observed that more than one half of patients use multiple medications, but many of them don’t feel satisfied with the symptoms scores [4]. Finally, since AR is considered a trivial disease, many patients do not seek medical care or specific diagnosis and primarily refer to pharmacies for self medications [5], or refer to alternative/complementary medicines [6], whereas allergen specific immunotherapy (AIT) is neglected [7]. Currently, topical/systemic antihistamines and intranasal corticosteroids are considered the first-line therapy, but many other treatments (such as decongestants, cromones or anti-muscarinic agents) are available over the counter.

Despite the social health costs [8], it emerges that generally AR is underestimated, often poorly controlled and undertreated. Thus, a more detailed education for healthcare workers and patients would be needed and a better awareness of the disease should be disseminated. It is true that, so far, there are no predictive biomarkers to appropriately address the therapeutic approach, or to predict the response, for instance, to AIT [9]. According to these considerations, in the last decade greater attention has been devoted to a more comprehensive approach to AR [10], looking specifically to the severity of symptoms, exacerbations, impact on the quality of life, course of disease, use of medications. The web-based instruments are a promising example of the possibility of day-by-day monitoring of patients [3].

Currently in real life the most feasible and practical instrument to evaluate the presence, severity and control of symptoms remains the Visual Analogue Scale (VAS), consisting in a single patient-reported outcome of the effect of the disease and of the treatment. Patients with a VAS > 5 are considered not controlled [11–13]. Accordingly, most patients agreed that VAS evaluation could be considered a good instrument [14, 15].

Other instruments to assess the impact of AR are available, for example the CARAT (“Control of Allergic Rhinitis and Asthma Test”) questionnaire evaluates in few questions the perceived control of AR and concomitant asthma, also assessing the overall use of pharmacotherapy [16]. Other more detailed questionnaires are also available. It has been identified that the major reasons for an unsatisfactory control of rhinitis are incorrect diagnosis, intrinsic severity of the disease [17], incorrect use/misuse of the intranasal treatment [11].

The aim of this study was to assess through the use of VAS and CARAT instruments the level of control of AR in patients (> 16 years) without previous asthma diagnosis referred to Allergy Centers. The possible causes of poor control were also further investigated.

## Methods

This was a multi-center observational cross-sectional study involving patients with AR, referred for the first time to Allergy centers of Northern Italy (Turin, Verona, Parma, Brescia) between May and December 2015. Only patients with ascertained symptoms of AR in the previous month and without a previous asthma diagnosis were included. The level of control was assessed by the VAS (0 = troublesome symptoms, 10 = no symptoms) and the CARAT instrument [16]. CARAT is a tool created and validated to measure disease control of both allergic rhinitis and asthma. It is a self-administered questionnaire that quantifies not only nasal, ocular, oropharyngeal, lower respiratory tract symptoms, sleep impairment, activities, psychosocial impediments, but also treatment and exacerbation. The more the patient is symptomatic and has a poor quality of life, the lower the CARAT score. In addition, the relevant demographic and clinical data (age, sex, duration of the disease, allergen sensitizations) were recorded for the analyses. The type of medications (e.g. antihistamines, nasal steroids, decongestants), their frequency of dosing and possible incremental use were assessed. The AR was better controlled when the VAS was lower and the CARAT scores were higher.

All statistical procedures were performed using the statistical package STATA version 14 for Windows [STATA^®^ (Stata Corp-LP-College Station-TX-USA)] [18]. All tests of significativity were carried out at a 0.05 level. For the analysis of symptom severity and symptomatic treatment changes, the ratings “present always, never, less than or more than 2/week” were used. Age was categorized in 10-year intervals. The time of symptoms’ duration was classified as < 1, 1–4, 5–10, 11–20, > 20 years. All categorized variables were used in the analysis as ordinal variables when appropriate. The relationship between VAS and symptoms score was measured by means of quantile (median) regression with VAS-score as dependent and Symptom-score as predictive variables [19–21].

Statistical methods were used to investigate which factors could influence the VAS score and the relationship between VAS score (dependent variables) and symptoms, medication use and life quality.

Ordinal logistic regression with VAS score as dependent variable, measuring the increase in the log odds of being in a higher level of VAS-score for a class increase in each predictive variable (i.e., going from 0 to 4), given all the other variables in the model are held stable. The Parallel Regression Assumption was tested by Brant Test [20, 21]. The same analysis was repeated by means of binomial logistic regression categorizing VAS score as < 5 or ≥ 5. The predicted marginal probabilities of VAS ≥ 5 (in % scale) were computed and reported by age strata. To assess the influence of category of drug used on the probability of VAS > 5 we performed logistic model analysis. In all analysis bootstrap estimation of standard error was used.

## Results

### Symptoms and treatment

Within the considered period, 250 patients (54% female, age between 16 and 80 years, 2% over 65 years) were studied (Table 1). Overall 45% of patients had symptoms of AR for less than 5 years, and 12% of the patients reported a persistence of symptoms greater than 20 years. Most patients reported no comorbidity associated with rhinitis and among patients with comorbid conditions 20.8% had nasal polyposis and 2.6% had an acetylsalicylic acid-exacerbated disease. 79% were polysensitized, mainly to grass (68.4%), house dust mites (38%) and cat dander (31.6%). In 52% of the cases the most frequently reported symptom was nasal obstruction (daily or > 2/week). 29% of patients reported mild obstruction, whereas 18% had no obstruction during the previous 4 weeks. The second relevant symptom was rhinorrhea, frequent in 50%, mild in 33% and absent in 17%. Sneezing and itching were overall less frequent. According to the ARIA guidelines [1], 34% patients reported a relevant impact of AR on their daily activities. Nocturnal awakenings were reported by 52% of patients (Table 2).

**Table 1:** Demographic data of the population

Sex	
Male	115/250 (46%)
Female	135/250 (54%)
Age range (years)	
10–20	45/250 (18%)
20–45	143/250 (57%)
45–65	57/250 (23%)
> 65	5/250 (2%)
Onset of symptoms (years)	
1–5	113/250 (43%)
5–10	37/250 (15%)
10–20	70/250 (28%)
> 20	30/250 (12%)
Allergen sensitization	
Monosensitized	52/250 (21%)
Polysensitized	198/250 (79%)

**Table 2:** Symptoms frequency

Nasal obstruction	
Never	45/250 (18%)
1–2/week	73/250 (29%)
> 2/week	72/250 (29%)
Always	59/250 (23%)
Rhinorrhea	
Never	42/250 (17%)
1–2/week	82/250 (33%)
> 2/week	74/250 (29%)
Always	52/250 (21%)
Sneezing	
Never	34/250 (14%)
1–2/week	94/250 (38%)
> 2/week	66/250 (26%)
Always	56/250 (22%)
Itching	
Never	61/250 (24%)
1–2/week	89/250 (36%)
> 2/week	65/250 (26%)
Always	35/250 (14%)
Quality of life	
Never	77/250 (31%)
1–2/week	85/250 (34%)
> 2/week	70/250 (28%)
Always	18/250 (7%)
Awakenings	
Never	119/250 (48%)
1–2/week	68/250 (27%)
>2/week	53/250 (21%)
Always	10/250 (4%)

Within the population, 71% of patients (178/250) were assuming symptomatic drugs, such as antihistamines (68% as regular treatment and 32% on demand), nasal steroids (75% regular and 25% on demand), nasal lavages (14% on demand). 59% of the subjects taking any therapy did not report an increase in drug consumption for more than 1 week/month.

### Risk factors of severe disease

**Fig. 1:**
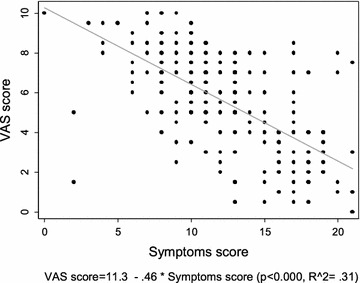
Median regression of VAS score versus symptoms-score

More importantly, 62% of the patients scored a VAS ≥ 5, suggesting a non-optimal control of the disease in the previous last 4 weeks. By the median regression, a negative weak correlation (Spearman’s rho = − 0.65 p < 0.01) was seen between VAS and symptom scores (Fig. 1). The ordinal logistic regression analysis showed that, controlling for other symptoms, the probability of an higher VAS-score level increases significantly with stuffy nose and sneezing symptoms (OR 2.06–CI 95% 1.57–2.71), quality of life compromised (OR 1.64 CI 95% 1.17–2.28) and night awakenings (OR 1.44 CI 95% 1.03–2.00) (Table 3). In those patients with uncontrolled AR, nasal obstruction resulted to be the most relevant symptom.

**Table 3:** Risk of higher VAS-symptoms score by QoL and CARAT items score adjusted each-other and for age by means of ordinal logistic regression)

VAS-score	Odds ratio	95% CI		p value
Age (media 34 years old)	0.98	0.97	1.00	0.020
Nasal obstruction (204/250)	2.06	1.57	2.71	0.000
QoL (169/250)	1.64	1.17	2.28	0.004
Awakenings (131/250)	1.44	1.03	2.00	0.031
Itching (189/250)	1.20	0.91	1.58	0.191
Drug consumption (178/250)	1.22	0.80	1.86	0.349

**Fig. 2:**
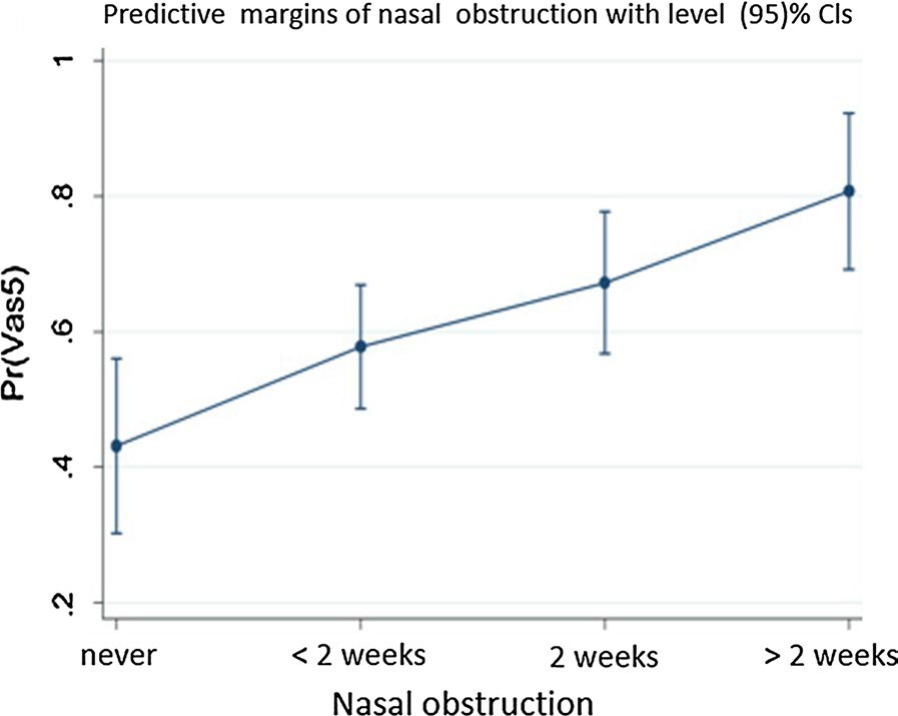
Probability of VAS score > 5 by nasal obstruction frequency, adjusted for other CARAT items by means of logistic regression analysis

The risk of uncontrolled disease is associated considering the probability of having VAS score > 5 (as lack of control of disease) the logistic regression analysis showed that itching and nocturnal awakenings have less influence on the control of AR (Table 4) than nasal obstruction and sneezing frequency. The frequency of nasal obstruction in linearly associated with VAS score > 5, (OR 1.78 for each nasal obstruction frequency grade, CI 95% 1.24–2.55) (Fig. 2). There was no significant association between control of the disease and demographic characteristics (age, sex, comorbidities, duration of the disease).

**Table 4:** Risk of VAS > 5—uncontrolled disease—by level of CARAT item (categorical) controlling each-other

	Odds ratio	95% Confidence interval			p value
Nasal obstruction					
Never	2.04	0.74–5.60			0.168
1–2/week	3.59	1.21–10.63			0.021
> 2/week	6.76	1.87–24.50			0.004
Linear trend	1.78	1.24–2.55			0.002
Sneezing					
Never	0.32	0.10–1.03			0.056
1–2/week	1.34	0.37–4.88			0.655
> 2/week	6.84	1.17–40.11			0.033
Linear trend	1.99	1.27–3.13			0.003
Itching					
Never	3.43	1.30–9.05			0.013
1–2/week	2.58	0.83–8.09			0.103
> 2/week	3.21	0.60–17.22			0.173
Linear trend	1.52	0.99–2.33			0.053
Awakenings					
Never	1.44	0.59–3.50			0.421
1–2/week	3.63	1.09–12.03			0.035
> 2/week	2.38	0.20–28.40			0.493
Linear trend	1.67	1.04–2.69			0.034

Result of logistic regression analysis using VS > 5 as dependent and CARAT Items as predictive variables

### Treatment of rhinitis

In patients with uncontrolled disease (VAS > 5), oral antihistamines were the most used medications (p = 0.005), whereas nasal steroids (as suggested by ARIA guidelines) were less used (p = 0,604) and allergen immunotherapy was the least employed (p = 0.037) (Table 5). In patients who needed to increase therapy (23/178), the most used drugs were steroids, both topical and systemic, and the steroid/antihistamine topical combination.

**Table 5:** Risk of VAS > 5—uncontrolled disease—by therapeutic approaches used in patients

	Odds ratio		95% CI	p value > z
Antihistamines	2.33	1.30	4.20	0.005
AIT	0.37	0.15	0.94	0.037
Nasal steroids	0.82	0.38	1.76	0.604
Antihistamines plus nasal steroids	0.74	0.16	3.52	0.704
Nasal lavage	1.32	0.60	2.91	0.485
Systemic steroids	0.48	0.13	1.82	0.28

## Discussion and conclusions

It is well known that AR is considered as a “trivial” disorder in the general population. Nonetheless, it probably remains the most common immune-mediated disease, with prevalence continuously increasing, and being responsible for a not negligible economic burden in term of absenteism or presenteeism [22, 23]. It has recently become clear that, despite the easy diagnosis, AR remains often uncontrolled or inappropriately treated. This can have a reflection on the natural history of the disease, as AR is the most relevant risk factor for the future development of allergic asthma [24–26]. In this multi-center cross-sectional study, conducted in Northern Italy, we aimed at evaluating the level of control of AR by means of a simple tool, the VAS, that can be easily filled by patients and reflects the presence of symptoms and their burden in the previous month. A VAS score > 5 suggests a not satisfactory control of symptoms. This parameter was correlated with CARAT, single symptoms, and the consumption of AR-related drugs. More than 60% of the patients analysed had an uncontrolled AR, providing evidence in line with the available literature data [4]. A European survey evidenced that a satisfactory control of AR symptoms can be achieved only in about 45% of the patients, independently of the drugs used [23]. It is also true that in our survey, including selected patients, only about one half was using multiple medications, and that only a minority was undergoing AIT. This survey confirmed that the VAS assessment is a reliable clinical tool, as it well correlates with the structured CARAT questionnaire and with the relevant symptoms of AR. In our study, nasal obstruction, the quality of life and nocturnal awakenings significantly impacted on the VAS, but the best index of the failure to control was the presence of nasal obstruction. Even this observation conforms to what can be found in the literature, as nasal obstruction is the most difficult symptom to treat being it reported by most troublesome patients. This finding suggests to always evaluate this symptom as a predictive marker of poor control of the disease. We could not show any correlation between the lack of AR control and asthma, since our patients were selected without asthma and in a cross-sectional fashion [18, 19]. Finally, the most alarming fact that emerges is that the ARIA guidelines are still poorly followed. In fact, our AR patients, even if not controlled, mainly used antihistamines but not nasal steroids. Also, AIT is poorly used in our sample, although this observation could be biased by the fact that the most part of patients was at the first allergologic visit. Our series included patients who came after the evaluation of general practitioners, post-Emergency Room or specialists (ENT, paediatricians, pulmonologists). This suggests that not only general practitioners, but also other medical categories ignore or do not follow the ARIA guidelines, which must therefore have a greater circulation in order to obtain a better control of allergic rhinitis.
